# A curious case of cysteines in human peroxiredoxin I

**DOI:** 10.1016/j.redox.2020.101738

**Published:** 2020-09-24

**Authors:** Ashu Mohammad, Reena V. Saini, Rakesh Kumar, Deepak Sharma, Neeraj K. Saini, Arpit Gupta, Priyanka Thakur, Christine C. Winterbourn, Adesh K. Saini

**Affiliations:** a Department of Biotechnology and Central Research Cell, MMEC, Maharishi Markandeshwar (Deemed to be University), Mullana, Ambala, Haryana, 133207, India; b Faculty of Applied Science and Biotechnology, Shoolini University, Solan, 173229, India; c Council of Scientific and Industrial Research-Institute of Microbial Technology, Chandigarh, India; d Department of Biotechnology, Jawaharlal Nehru University, Delhi, 110067, India; e Faculty of Sciences, Shoolini University, Solan, 173229, India; f Centre for Free Radical Research, Department of Pathology and Biomedical Science, University of Otago, Christchurch, New Zealand; g Maharishi Markandeshwar (Deemed to Be University), Solan, HP, 173212, India

**Keywords:** Human peroxiredoxins, Catalytic residues, Yeast, Redox stress, Chaperone activity, *Saccharomyces cerevisiae*, hPrx, human peroxiredoxin, Tsa, thiol specific antioxidant, H_2_O_2_, hydrogen peroxide, SNP, Sodium nitroprusside, ROS, reactive oxygen species, RNS, reactive nitrogen species, Cys, Cysteine, Srxs, sulfiredoxins, HMW, high molecular weight

## Abstract

Peroxiredoxins (Prxs) are antioxidant proteins that are involved in cellular defence against reactive oxygen species and reactive nitrogen species. Humans have six peroxiredoxins, hPrxI-VI, out of which hPrxI and hPrxII belongs to the typical 2-Cys class sharing 90% conservation in their amino acid sequence including catalytic residues required to carry out their peroxidase and chaperone activities. Despite the high conservation between hPrxI and hPrxII, hPrxI behaves differently from hPrxII in its peroxidase and chaperone activity. We recently showed in yeast that in the absence of Tsa1 and Tsa2 (orthologs of hPrx) hPrxI protects the cells against different stressors whereas hPrxII does not. To understand this difference, we expressed catalytic mutants of hPrxI in yeast cells lacking the orthologs of hPrxI/II. We found that the catalytic mutants lacking peroxidase function including hPrxI^C52S^, hPrxI^C173S^, hPrxI^T49A^, hPrxI^P45A^ and hPrxI^R128A^ were not able to grow on media with nitrosative stressor (sodium nitroprusside) and unable to withstand heat stress, but surprisingly they were able to grow on an oxidative stressor (H_2_O_2_). Interestingly, we found that hPrxI increases the expression of antioxidant genes, *GPX1* and *SOD1,* and this is also seen in the case of a catalytic mutant, indicating hPrxI can indirectly reduce oxidative stress independently of its own peroxidase function and thus suggesting a novel role of hPrxI in altering the expression of other antioxidant genes. Furthermore, hPrxI^C83T^ was resistant to hyperoxidation and formation of stable high molecular weight oligomers, which is suggestive of impaired chaperone activity. Our results suggest that the catalytic residues of hPrxI are essential to counter the nitrosative stress whereas Cys83 in hPrxI plays a critical role in hyperoxidation of hPrxI.

## Graphical abstract

**Figure:**
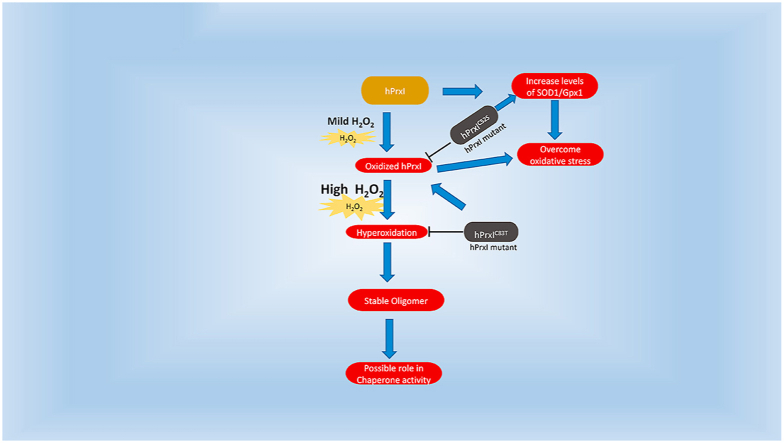

## 1 Introduction

Peroxiredoxins (Prxs) are ubiquitous antioxidant proteins that utilize cysteine (Cys) residues to reduce peroxides and regulate redox signalling within the cells [[1], [2], [3]]. Multiple Prx isoforms are present in different species, for example five in S*accharomyces cerevisiae* and six in humans [4]. In humans, these isoforms (abbreviated here as hPrx) are distributed in different parts of a cell. For instance, in humans hPrxI and hPrxII are mainly present in the cytoplasm and hPrxIII in the mitochondria.

The conserved *N*-terminal peroxidatic Cys (C_P_-SH) residue of Prxs reacts with peroxides to form C_P_-SOH (sulfenic acid) which then forms a disulfide bond. Based on the mechanism to form the disulfide, Prxs can be divided into three sub-groups: typical 2-Cys Prxs, atypical 2-Cys Prxs and 1-Cys Prxs. In the typical 2-Cys Prxs, the sulfenic acid forms an intersubunit disulfide bond with a C-terminal resolving Cys (C_R_-SH) residue of the other subunit in the Prx homodimer [5]. In the case of atypical 2-Cys Prxs, the sulfenic acid forms an intrasubunit disulfide bond and, in 1-Cys Prxs, where the resolving Cys is absent, the sulfenic acid forms the disulfide bond with GSH and possibly other proteins carrying a thiol group. The disulfides are then recycled, for typical Prxs predominantly by thioredoxin/thioredoxin reductase. But if peroxide levels are high, C_P_-SOH gets overoxidized to the inactive sulfinic acid (C_P_-SO_2_H) and sulfonic acid (C_P_-SO_3_H) forms. These forms can be restored to the reduced thiolate by sulfiredoxins (Srxs) in the presence of ATP and Mg^2+^ [6]. Both the hyperoxidized and reduced forms of hPrxI make a high molecular weight (HMW) decameric complex but the hyperoxidized form gives a more stable decameric form than the reduced, possibly due to redox-regulated conformational changes in the conserved GGLG and YF motifs of hPrxI as predicted earlier [1]. These motifs make Prxs more sensitive to hyperoxidation and play a crucial role in its shift to stable HMW decameric complexes which then leads to increased chaperone activity [[7], [8], [9]]. In humans, both hPrxII and hPrxIII have these conserved motifs but interestingly their sensitivity to hyperoxidation is different. hPrxII is more sensitive to hyperoxidation than hPrxI whereas hPrxIII is less sensitive, possibly due to the presence of other key residues at the C-terminal [[10], [11], [12], [13]].

In our previous paper we investigated the role of oxidation state of hPrxI and hPrxII in overcoming redox stress. Both appeared capable of undergoing redox cycling in yeast, which is as expected from the similarity of the active site region of human and yeast thioredoxins [[14], [15], [16]] and the reported ability of the yeast thioredoxin system to reduce all the human 2-Cys Prxs [17]. However, hPrxI and hPrxII showed different behaviour towards stressors when expressed in *tsa1tsa2Δ* yeast strain [18]. Herein, we investigated the role of catalytic and non-catalytic residues of hPrxI in countering oxidative and nitrosative stress using the yeast model. So, we mutated hPrxI to replace catalytic Cysteine residues with Serine/Alanine. The catalytic mutants of hPrxI which were unable to form disulfide-linked dimers were sensitive to nitrosative stress, but surprisingly survived oxidative stress. Our results also indicate that the extra Cys present in hPrxI i.e. C83, has an important role in oligomerization which could be linked to the role of hPrxI in chaperone activity.

## 2 Materials and methods

### 2.1 Chemicals

Restriction enzymes, Taq polymerase and ligase were purchased from New England Biolabs Inc, USA. Reagents and protein standards were obtained from Sigma-Aldrich if otherwise not stated. Antibodies were from Abcam (*anti*-PrxI), Sigma (*anti*-PrxII), and Abfrontier Seoul Korea (*anti*-PrxSO_2/3_) Santa Cruz Biotechnology (anti-rabbit secondary antibody) and Thermo Fisher Scientific (*anti*-Pgk1). Protease inhibitor cocktail (PIC) was from Thermo Fisher Scientific. Solvents were obtained from Thermo Fisher Scientific.

### 2.2 Cloning and mutagenesis

The pYEURA3 shuttle vector carrying *hPRXI* [18] was used as template to create desired mutations in catalytic and non-catalytic residues using forward and reverse primers. The clones were confirmed after sequencing and then these plasmids carrying mutations were transformed in *Saccharomyces cerevisiae* strain (*MAT*a *his3*Δ*1 leu2*Δ*0 met15*Δ*0 ura3*Δ*0 tsa1::KAN tsa2::LEU2)* lacking *tsa1* and *tsa2* genes (*tsa1tsa2Δ*). Isogenic WT strain (BY4741) [19] containing intact *TSA1* and *TSA2* and, *tsa1tsa2*Δ strain were transformed with pYEURA3 vector [19], respectively.

### 2.3 H_2_O_2_ and SNP sensitivity assays

Effects of oxidative (H_2_O_2_) and nitrosative stress (sodium nitroprusside; SNP) were determined by growing the yeast cells to mid-log phase in selective drop out (SD) media without uracil (SD-U) and ten-fold serial dilutions were spotted onto SD-U and SD-U media containing H_2_O_2_ or SNP. The cells were incubated at 30 °C for 2 days (SD-U) and 4 days (SD-U containing H_2_O_2_ or SNP).

### 2.4 Analysis of hyperoxidized and dimeric hPrxI

Cells were grown at 30 °C as described above and 1 OD_600_ cells were treated with H_2_O_2_ for 1 h. Then cells were harvested, washed with phosphate buffered saline (PBS) and incubated with PBS containing 50 mM *N*-ethylmaleimide (NEM) on ice for 30 min to block the total free thiols. Cells were lysed by vortexing with autoclaved glass beads using lysis buffer (0.2% Triton X-100, 100 μg/ml PMSF, 1*X* (PIC), 1 mM EDTA and 50 mM NEM in PBS). Total soluble proteins were separated by centrifugation and mixed with non-reducing or reducing (1% β-mercaptoethanol) Laemmli buffer. The samples were resolved on 12% SDS-PAGE gels and immunoblot analysis was conducted as described [3,20] using antibodies against hPrxI, hPrxII, hPrx-SO_2/3_ and, Pgk1 (as a control).

### 2.5 Confocal microscopy

For monitoring the presence of ROS inside the yeast cells 2′,7′-dichlorofluorescein diacetate (DCFDA; Sigma–Aldrich) was used [21]. Cells were grown in SD-U media till OD_600_ 1, treated with H_2_O_2_ for 15 min and then incubated with DCFDA for 10 min in the dark. The cells were washed with the same media and were mounted onto 1% Agarose blocks and imaging was done using 60*X*/oil objective, with a numerical aperture of 1.45. The 2′,7′-dichlorofluoresecin (DCF) was excited with argon laser and a helium-neon laser (λ_em_ = 529 nm λ_exc_ = 488 nm) using Nikon A1plus Ti confocal microscope with Nikon A1R scan head. Images were captured using NIS elements software.

### 2.6 Quantitative real time PCR (qRT-PCR)

Quantitative real time PCR was used to measure the mRNA transcripts of *SOD1* and *GPX1*. *PGK1* (phosphoglycerol kinase) was selected as reference gene. The total RNA from cells treated with or without H_2_O_2_ was isolated using HiPurA Yeast RNA purification kit (HiMedia-MB611) following manufacturer's protocol. The cDNA was prepared from isolated RNA using cDNA synthesis kit (iScript DNA synthesis kit Bio-Rad-1708891) and used as template for qRT-PCR. cDNA samples were amplified using iTaq Universal SYBR Green Supermix (Bio-Rad) on CFX96Real Time PCR system (Bio-Rad). Using the software GraphPad-prism, the statistical significance (p value ≤ 0.05) was measured using one-way ANOVA followed by Tukey's post-test [22].

### 2.7 Size exclusion chromatography (SEC)

Total yeast cell lysate was prepared from 100 ml of 1.0 OD_600_ cells after treatment with or without H_2_O_2_. Total clarified lysate was filtered with 0.45 μ filter and then injected on to a Superdex 10/200 SEC column (GE Healthcare) previously equilibrated with PBS. Selected lysate fractions were resolved on 12% reducing SDS-PAGE and hPrxI was analysed by immunoblotting using anti hPrxI antibody.

## 3 Results and discussion

### 3.1 Catalytic residues of hPrxI are critical to overcome nitrosative stress but not for oxidative stress

In this study our aim was to dissect the role of different conserved residues in hPrxI for overcoming oxidative and nitrosative stress using the yeast model system. In previous studies, we and others found that hPrxI and not hPrxII, could complement the defects induced in *tsa1tsa2Δ* mutant, suggesting that human hPrxI is an orthologue of yeast Tsa1 [4,18]. In these studies, hPrxI and hPrxII were expressed in yeast using the *TSA1* promoter, which was shown to give protein expression levels of hPrxI and hPrxII equivalent to yeast Tsa1 [4,18]. Using the same strategy, we also found that there is a difference in the behaviour of hPrxI and hPrxII, despite having 90% conservation in their amino acid sequence [4]. Using the yeast model system, we also showed that a more reducing intracellular redox state of the hPrxs reflected a greater ability to overcome oxidative stress [18]. To understand the role of hPrxI catalytic residues in overcoming redox stress, we introduced a series of mutations in the *hPRXI* gene using site-directed mutagenesis. Mutations were confirmed by DNA sequencing. The yeast plasmid carrying mutation in *hPRXI* was transformed into the strain deleted for both *TSA1* and *TSA2* (referred herein as *tsa1tsa2Δ*) (*tsa1::kan, tsa2::leu*) a derivative of BY4741 yeast strain (*MATa, his3Δ1, leu2Δ0, met15Δ0, ura3Δ0*) [19]. The generated strains were grown on SD-U media and serial dilutions were spotted on the same with or without the stressors i.e. H_2_O_2_ or SNP. The selected concentration of stressors was based on our previous study [18]. As replicated in Fig S1, the *tsa1tsa2Δ* strain grew more slowly in the absence of stress and this was overcome by hPrxI or PrxII (Fig S1A), whereas the slower growth under oxidative (Fig S1B) or nitrosative stress (Fig S1C) could only be overcome by hPrxI.

**Fig. 1:**
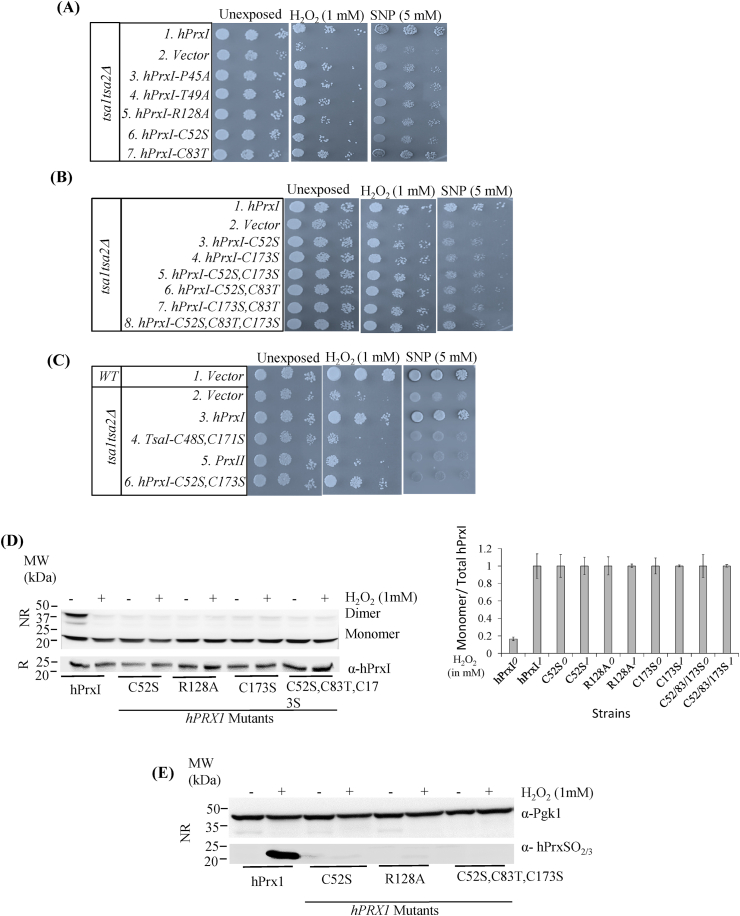
**Catalytic residues in hPrxI that are required for disulphide formation are indispensable to overcome nitrosative stress but not for oxidative stress in yeast.** (A) *tsa1tsa2Δ* strain carrying pYEURA3 alone, pYEURA3 expressing hPrxI or hPrxI^C52S^ or hPrxI^C83T^ or hPrxI^P45A^ or hPrxI^T49A^ and hPrxI^R128A^ were grown to OD_600_ 1 in SD-U. Ten-fold serial dilutions (undiluted, 10*X* and 100*X* diluted) were spotted on SD-U media without any stress i.e. unexposed or with stressors, 1 mM H_2_O_2_ or 5 mM SNP. The cells were incubated at 30 °C for 2 days for unexposed or 4 days for 1 mM H_2_O_2_ and 5 mM SNP. The results shown for spotting assays are typical of three biological repeats. (B) *tsa1tsa2Δ* strain carrying pYEURA3 alone or *hPRXI* or hPrxI^C52S^ or hPrxI^C173S^ or hPrxI^C52S,C173S^ or hPrxI^C173S, C83T^ or hPrxI^C52S, C83T^ or hPrxI^C52S,C83T,C173S^ were spotted with same method and in same plates as discussed earlier in A. (C) *BY4741* strain carrying pYEURA3 vector, *tsa1tsa2Δ* strain carrying pYEURA3 alone, pYEURA3 carrying *TSA1*^*C48S,C171S*^ or *hPRXI* or *hPRXII* or *hPrxI*^*C52S,C173S*^ were spotted with same method and in same plates as discussed earlier. (D) Vector, hPrxI, hPrxI^C52S^, hPrxI^C173S^, hPrxI^C52S,C83T,C173S^ and hPrxI^R128A^ strains were grown to OD_600_ 1 and treated with 1 mM H_2_O_2_ for 1 h at 30 °C. Cells were treated with 50 mM NEM in PBS for 30 min on ice. Cell lysates were prepared in the presence of 50 mM NEM without (NR; non-reducing) or with 1% β-mercaptoethanol (R; reducing) and resolved using SDS-PAGE and immunoblotting was done using *anti*-hPrxI antibody. Locations of dimer and monomer are shown. Bands of hPrxI by immunoblotting were quantified by ImageJ (NIH) software and the ratio of hPrxI monomer and total hPrxI were calculated. Data are mean and range from two independent experiments. (E) Cells were treated as in (A), and non-reducing gel was probed with *anti*-PrxSO_2/3_ antibody to detect hyperoxidation of hPrxI or with *anti*-Pgk1 for analyzing the loading of proteins.

The two key cysteine residues of hPrxI are C52 and C173, where C52 is the peroxidatic cysteine that reduces peroxides and C173 is the resolving cysteine that forms a dimer with C52 [[23], [24], [25]]. P45, T49 and R128 are involved in activating the peroxide and are critical for its high reactivity [1]. Mutations in these residues were analysed for their impact on the growth of the yeast strain under stress conditions (1 mM H_2_O_2_ or 5 mM SNP). As compared to the wild-type hPrxI, none of the active site mutants, hPrxI^C52S^, hPrxI^C173S^, hPrxI^P45A^, hPrxI^T49A^ and hPrxI^R128A^, grew on the media containing SNP indicating that these residues were critically involved in overcoming the nitrosative stress (Fig. 1A compare lines 1 and 3–6 and 1B compare lines 1 and 3–4). In the presence of SNP, the growth pattern of these mutant strains, as demonstrated in the spot intensities on serial dilution, was similar to the strain carrying empty vector control (Fig. 1B compare lines 2 and 3–8). These cells grew poorly compared with the cells expressing hPrxI or the wild type strain expressing Tsa1 and Tsa2 (Fig. 1C compare lines 1,3 and 6). Similar to the catalytic mutants of hPrxI, the Tsa1^C48S,C171S^ mutant also showed no growth on media containing SNP (Fig. 1C compare lines 1 and 4). The growth defects on SNP media are not due to a decrease in the protein stability owing to mutation because the steady-state expression levels of the mutant proteins were found to be similar to that of WT-PrxI (Fig. 1D).

Very surprisingly, the catalytic mutants, hPrxI^C52S^, hPrxI^C173S^, hPrxI^P45A^, hPrxI^T49A^ and hPrxI^R128A^ were all able to grow on media containing the oxidative stressor H_2_O_2_ (Fig. 1B compare lines 1 and 3–4 and 1A compare lines 1 and 3–6). The growth pattern of these mutants was similar to that of WT-hPrxI. We further created the double mutant (hPrxI^C52S,C173S^) and found results similar to the single catalytic mutants (Fig. 1B compare lines 1 and 3–5). The phenotypic results indicated that the catalytic residues were not essential for overcoming the oxidative stress.

Since hPrxI contains another cysteine, at 83 position, which is absent in hPrxII, we created hPrxI^C83T^ and combinations of double hPrxI^C173S, C83T^, hPrxI^C52S, C83T^, and a triple hPrxI^C52S,C83T,C173S^ mutant and expressed them in *tsa1tsa2Δ* yeast strain. We found that hPrxI^C83T^ show no effect on oxidative and nitrosative stress (Fig. 1A compare lines 1 and 7). Also, when Cys83 was mutated along with the active site mutations, these strains were able to grow on H_2_O_2,_ but none grew on media containing SNP (Fig. 1B compare lines 1 and 6–8).

In addition to the spotting assay, we monitored growth of these mutants in the presence of stressors by recording their absorbance (OD_600_) at different time intervals. We found a similar trend in which the catalytic mutant hPrxI^C52S,C173S^ (Suppl. Fig. S1), hPrxI^C52S^, hPrxI^C173S^, hPrxI^P45A^, hPrxI^T49A^ and hPrxI^R128A^ (data not shown) showed growth kinetics similar to WT-hPrxI in unstressed cells (Fig. S1D) and in the presence of H_2_O_2_ (Fig S1E). In the presence of SNP, the catalytic mutants behaved like the vector control (Fig S1F). The hPrxI^C83T^ mutant showed similar growth characteristics to cells expressing WT-hPrxI (Fig S2).

As reported by Weids and Grant [26], we also found that the catalytic mutant of yeast tsa1^C48S,C171S^ was not able to grow on H_2_O_2_ and SNP (Fig. 1C compare lines 2 and 4). So, although it seems that hPrxI is a functional homologue of Tsa1, the genetic results indicate that they differ mechanistically, with Tsa1 but not hPrxI depending on the catalytic residues to overcome oxidative stress. As expected, and shown below, these hPrxI mutants cannot form disulfide-linked dimers and thus, cannot have peroxidase function. Peroxidase activity cannot therefore account for their ability to grow on media containing the oxidative stressor.

### 3.2 Catalytic mutants of hPrxI lack dimer formation but rescue oxidative stress phenotype

It is well known that the C52 and C173 residues in hPrxI are involved in the catalytic cycle. The thiol group (-SH) of C52 gets oxidized to sulfenic acid (-SOH) by H_2_O_2_ and forms a disulfide bond with C173 present on the other subunit [[23], [24], [25]]. This disulfide bond between two monomers results in the formation of a dimer. If the stress is high, the sulfenic acid (-SOH) gets hyperoxidized to the sulfinic (-SO_2_H) or sulfonic acid (-SO_3_H). These changes in the redox state of hPrxI can be detected in the yeast model by immunoblotting using specific antibodies [17]. To check for oxidation, the *tsa1tsa2Δ* cells expressing mutants of hPrxI were exposed to H_2_O_2_ for 1 h. Cell lysis was done in the presence of NEM to inhibit artefactual thiol oxidation and proteins in the lysates were resolved through non-reducing SDS-PAGE followed by immunoblotting. WT-hPrxI was partially dimerized even in untreated cells and became more monomeric after H_2_O_2_ exposure (Fig. 1D). As expected, hPrxI^C52S^ lacking the peroxidative Cys as well as hPrxI^C173S^ and the triple mutant hPrxI^C52S,C83T,C173S^ did not dimerize (Fig. 1D, upper NR panel). We also found that the hPrxI^R128A^ mutant (Fig. 1D) and the hPrxI^T49A^ mutant (not shown) did not form a dimer in yeast cells treated with H_2_O_2_, even though they contain the two Cys residues involved in dimer formation. This reflects their very slow reaction with H_2_O_2_ [27]. We further checked the effect of catalytic mutations on hyperoxidation by immunoblotting with an antibody that recognizes C52–SO_2/3._ In contrast to WT hPrxI, which was converted to the hyperoxidized monomer on exposure to H_2_O_2_, no hyperoxidation of hPrxI^C52S^, hPrxI^R128A^ and triple mutant hPrxI^C52S,C83T,C173S^ was detected (Fig. 1E).

**Fig. 2:**
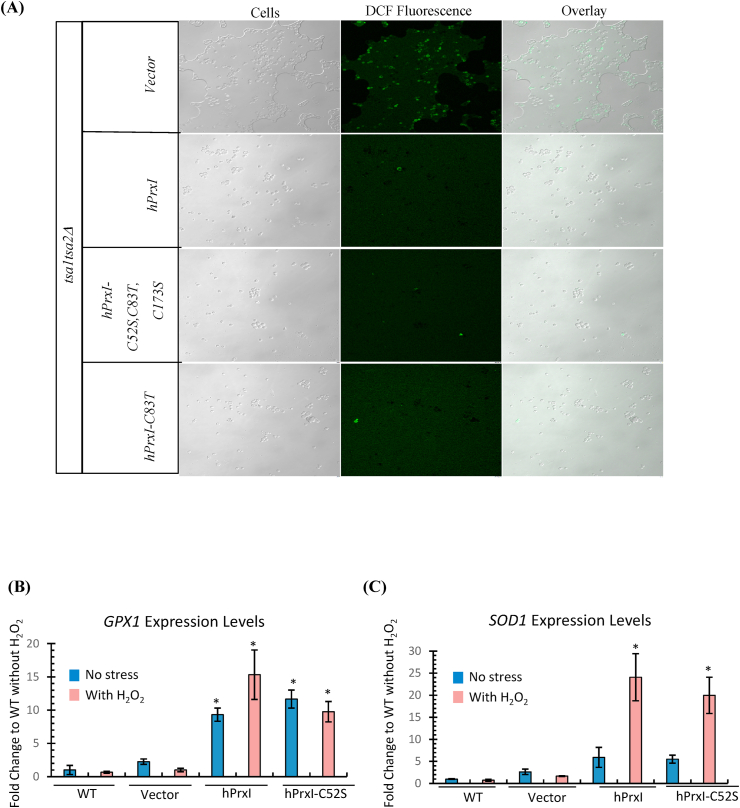
**Catalytic mutants can quench ROS in yeast and hPrxI increases the expression of *GPX1* and *SOD1.*** (A) Vector, hPrxI, hPrxI^C83T^ and hPrxI^C52S,C83T,C173S^ expressing strains were grown to OD_600_ 1 and treated with 1 mM H_2_O_2_ for 15 min and then incubated with DCFDA for 10 min in the dark. The cells were then washed with the same media and were mounted onto 1% Agarose blocks and analysed by confocal microscope. (B–C) WT, Vector, hPrxI and hPrxI^C52S^ strains were grown to OD_600_ 1 and treated with 1 mM H_2_O_2_ for 1 h. The total RNA was isolated from cell lysate and Quantitative real time PCR was used to measure the mRNA transcripts of the *GPX1* and *SOD1* with reference to *PGK1* levels. Values are means ± SEM of 3 biological replicates. Statistically significant, *p < 0.001 vs WT or vector.

Since the catalytic mutants of hPrxI were able to grow on H_2_O_2_, we wanted to establish if ROS levels were lower in these mutants. For this we used DCFDA which gets oxidized to a fluorescent dye 2′,7′-dichlorofluoresecin (DCF) [21]. Yeast cells treated with H_2_O_2_ were stained with DCFDA and analysed by confocal microscopy. There was no change in cellular morphology among the WT (carrying empty vector), vector (*tsa1tsa2Δ*) control, hPrxI and its mutants (Fig S3 and 2A). DCF fluorescence was highest in vector (*tsa1tsa2Δ*), whereas hPrxI^C83T^, hPrxI^C52S^ and WT-hPrxI showed similar levels of fluorescence (Fig. 2A). These results are in agreement with their growth on H_2_O_2_ stressors (Fig. 1A) and suggest that ROS levels were lower in the catalytic mutants even though their peroxidase activity was absent or very low.

### 3.3 hPrxI increases the expression of *SOD1* and *GPX1* in yeast

We further went on to explore the reason for the survival of cells expressing catalytic mutants of hPrxI on H_2_O_2_ stress. Apart from Prxs, other cellular antioxidants counter oxidative stress. It has been shown that yeast cells can adapt to increased ROS by overexpressing other antioxidant genes including *SOD1* and *GPX1* [28,29]. So, we measured the levels of *SOD1* and *GPX1* mRNA in WT, vector control, hPrxI and hPrxI^C52S^ cells by qRT-PCR. Compared to WT cells, the cells carrying vector showed no significant change in expression whereas those expressing hPrxI had significantly higher expression of *GPX1* (~9-fold), under unstressed conditions increasing to ~15- fold in H_2_O_2_-stressed cells (Fig. 2B). hPrxI expression caused only a 5-fold increase in SOD1 expression compared with WT cells, but upon H_2_O_2_ treatment, expression increased significantly by ~24-fold (Fig. 2C). Very similar patterns of expression were observed for the cells expressing hPrxI^C52S^ (Fig. 2B and C). These results suggest that one possible reason for hPrxI mutants growing well under oxidative stress is by increasing the expression of *SOD1* and *GPX1* genes which can compensate for the inactivation of the peroxidase function of hPrxI^C52S^.

### 3.4 Cys-83 residue of hPrxI is crucial for hyperoxidation-mediated oligomerization

Apart from their peroxidase function, peroxiredoxins are also known for their chaperone activity. The chaperone function of hPrxI is favoured by the formation of oligomers, which are more stable upon hyperoxidation of C52 [2,3,30]. Besides C52, *in vitro* studies have shown that the C83 residue of hPrxI is involved in stabilizing the decameric structure via C83–C83 disulfide bond formation at the dimer-dimer interface [23]. Therefore, we mutated C83 and studied its effect on growth on stressors as well as the ability of the expressed hPrxI to oligomerise. Apart from C83, we also mutated the fourth Cys at position 71.

**Fig. 3:**
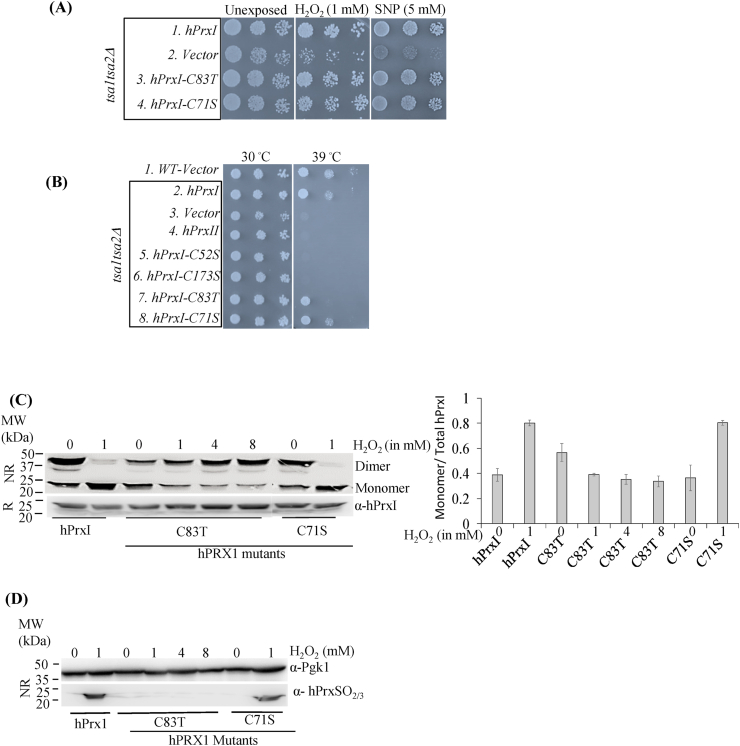
**hPrxI-C83 is important for hyperoxidation.** (A) Vector, hPrxI, hPrxI^C71S^ and hPrxI^C83T^ expressing strains were spotted with the same method and same plates as in Fig. 1A. (B) Vector, hPrxI, hPrxI^C52S^ or hPrxI^C173S^, hPrxI^C71S^ and hPrxI^C83T^ expressing strains were grown in SD-U media and were spotted on plates without any stressors but the incubation temperature was different. One plate was incubated at 30 °C for 2 days and the other plate was incubated at 39 °C for 4 days. The results shown for spotting assays are typical of three biological repeats. (C–D) Vector, hPrxI, hPrxI^C83T^ and hPrxI^C71S^ expressing strains were grown to OD_600_ 1 and treated with 1 mM H_2_O_2_ for 1h. Cells expressing hPrxI^C83T^ were also treated with 4 mM and 8 mM H_2_O_2_ for 1 h as indicated in the figure. Cells were treated with 50 mM NEM in PBS for 30 min on ice. Lysates were prepared as discussed in Fig. 1D, resolved in reducing and non-reducing gels and then immunoblotted with *anti*-hPrxI or *anti*-PrxSO_2/3_ to detect hyperoxidation of hPrxI or with *anti*-pgk1 antibody for analyzing the loading of proteins. Data are mean and range from two independent experiments.

We found that both hPrxI^C71S^ and hPrxI^C83T^ mutants were able to grow on media containing nitrosative and oxidative stress, indicating that C71 and C83 are not involved in overcoming these stresses (Fig. 3A compare lines 1 and 3–4; Fig. S2). To analyse their role in chaperone function, we monitored growth of the cells at 39 °C [31]. We found that hPrxI^C83T^ showed slower growth as compared to cells expressing wild-type hPrxI (Fig. 3B compare lines 2 and 7), whereas for hPrxI^C71S^ there was no difference (Fig. 3B compare lines 2 and 8). We also analysed the effect of hPrxI^C71S^ and hPrxI^C83T^ mutations on redox state (analysed by non-reducing SDS-PAGE) and sensitivity to hyperoxidation on exposing the cells to H_2_O_2_ stress (Fig. 3C&D). We found that, like wild-type hPrxI, hPrxI^C83T^ existed as a mixture of monomer and dimer in the absence of H_2_O_2_, but it was more resistant to oxidation by H_2_O_2_. It did not convert to the monomer (which is indicative of hyperoxidation) (Fig. 3C) and even with high concentrations (up to 8 mM) immunoblotting revealed no detectable hyperoxidation (Fig. 3D). hPrxI^C71S^ responded similarly to wild-type hPrxI. These results indicate that C71 is not required for protection against oxidative and nitrosative stress and thus does not affect growth on stressors. On the other hand, C83 seems to be important for hPrxI to undergo rapid hyperoxidation.

**Fig. 4:**
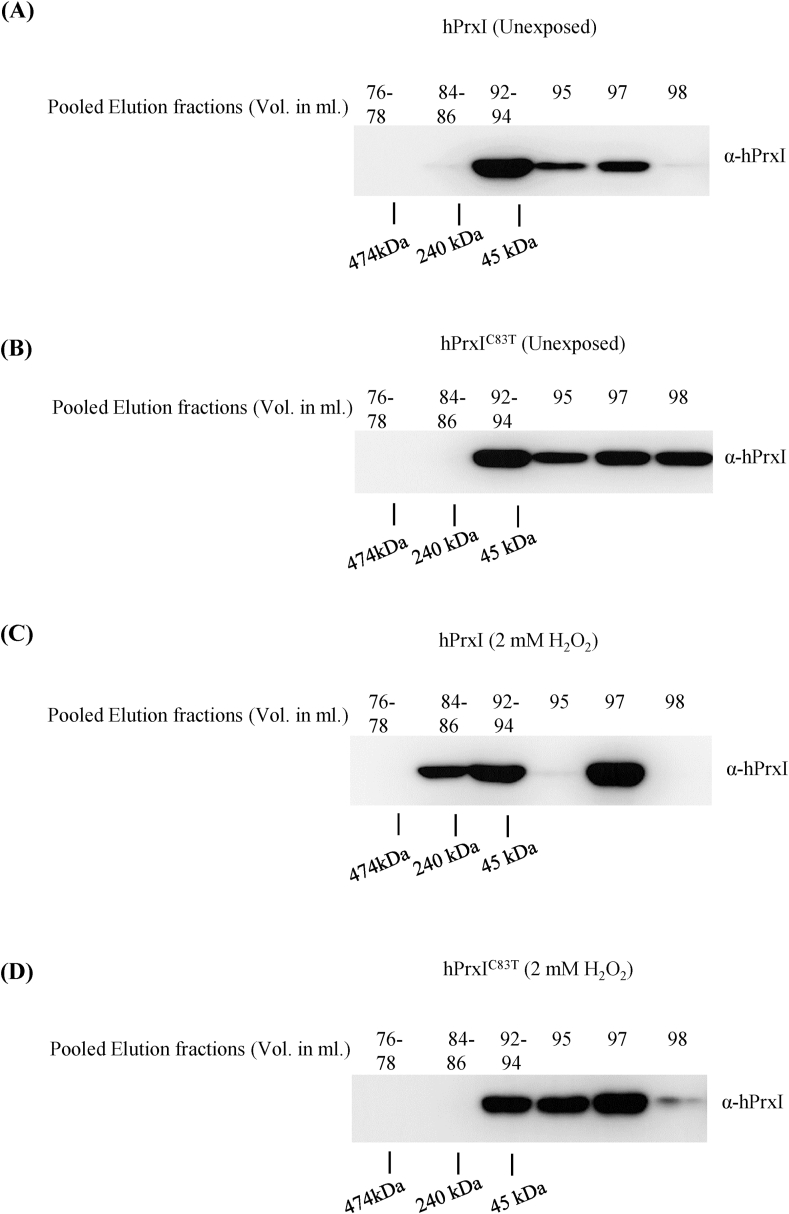
**hPrxI-C83 is crucial for hyperoxidation-dependent oligomerization.** (A–D) Yeast cells expressing hPrxI and hPrxI^C83T^ were grown to OD_600_ 1 and total yeast cell lysate was prepared from 100 mL. Total clarified lysate was filtered with a 0.45 μm filter and then 0.5 mL lysate was injected to superdex 10/200 SEC column (Column volume 80 mL and, Void volume 40 mL). The fractions of 1 mL were collected and numbered. The 76–78^th^ (3 mL), 84–86^th^ (3 mL), 92–94^th^ (3 mL), 95^th^, 97^th^ and 98^th^ fraction corresponding to the volume of eluant were concentrated and equal amount of protein from each were resolved on 12% reducing SDS-PAGE. hPrxI was analysed by immunoblotting using *anti*-hPrxI antibody. Protein standards: Ferritin- 474 kDa, Catalase- 240 kDa, Conalbumin- 76 kDa, Ovalbumin- 45 kDa, and RNase A- 13.69 kDa were run to estimate the molecular weight of proteins in lysate fractions. The results shown are typical of two biological repeats.

To confirm the role of Cys-83 in hyperoxidation-linked oligomerization we analysed the formation of oligomer/high molecular mass forms by performing size exclusion chromatography on cell lysates prepared from cells expressing *hPRXI* or *hPRXI*^*C83T*^ with and without H_2_O_2_ exposure. Collected fractions were resolved using SDS-PAGE and immunoblotting was done using *anti*-hPrxI. In untreated cell lysates, where WT hPrxI and *hPRXI*^*C83T*^ should be present predominantly as the disulfide form, both eluted later than the 240 kD marker in positions, consistent with the proteins being mainly dimeric (approximate size of hPrxI decamer is ~220 kDa and for dimer is ~45 kDa) (Fig. 4A&B). When the cells were treated with H_2_O_2_, hPrxI, which is hyperoxidized under these conditions (see Fig. 3D), underwent a partial shift to higher molecular weight oligomer (decamer) position (Fig. 4C). In contrast, *hPRXI*^*C83T*^, which was not hyperoxidized, did not undergo this shift (Fig. 4D). It is tempting to speculate that the resistance to hyperoxidation *hPRXI*^*C83T*^ and consequent lack of formation of high molecular mass oligomers, compromises its chaperone function [2,7,30,32] and this might account for the slow growth of the cells under heat stress as shown above.

## 4 Conclusion

In the present study we used a yeast model system to understand the role of catalytic residues in human PrxI to overcome the redox stress. Our results suggest that hPrxI requires its catalytic residues to overcome nitrosative stress. However, catalytic activity was not required to protect against oxidative stress. WT and all the catalytic mutants increased the expression levels of two important antioxidant genes *SOD1* and *GPX1* in yeast, and we hypothesise that this contributes to their protective effect. One possible mechanism, which needs to be confirmed, is that hPrxI independently of its catalytic residues activates transcription factors such as YAP1. YAP1 has been shown to act on antioxidant genes including *TSA1*, *SOD1*, *SOD2*, *CTT1*, and *TRX2* [28,29]. In principle, an increase in level of *GPX1* would provide an alternative enzymatic mechanism for removing H_2_O_2_. A protective effect of removing superoxide radicals with SOD1 is less obvious, but its expression may reflect a general response of the cells to counteract oxidative stress. The requirement for active hPrxI to protect against SNP could indicate that induced antioxidants like Sod1 and Gpx1 are less effective against nitrosative stress. However, further investigation is required to determine the significance of this mechanism. Another important finding of this study is that C83, the extra Cys in hPrxI, is an important determinant of its high reactivity with H_2_O_2_ and its susceptibility to hyperoxidation. Formation of stable decamers has been suggested to be crucial for chaperone activity. This activity, possibly in association with heat shock proteins like Hsp70 and Hsp104, could explain why WT PrxI was better than the C83T mutant at improving growth of yeast under heat stress. In conclusion our yeast system has provided a useful model for identifying unexpected mechanisms whereby hPrxI senses oxidative stress, by increasing expression of other antioxidant genes and exhibiting chaperone activity. Further investigation is warranted to determine the relevance of these mechanisms in mammalian cells where PrxI is naturally expressed.

## Declaration of competing interest

The authors declare that there are no conflicts of interest.
