# Supplementary material for: A curious case of cysteines in human peroxiredoxin I
Source: Redox Biol. 2020 Sep 24;37:101738. doi: 10.1016/j.redox.2020.101738 (PMC7530344; doi:10.1016/j.redox.2020.101738)
Supplement: Multimedia component 1 [file mmc1.pptx]

## Slide 1
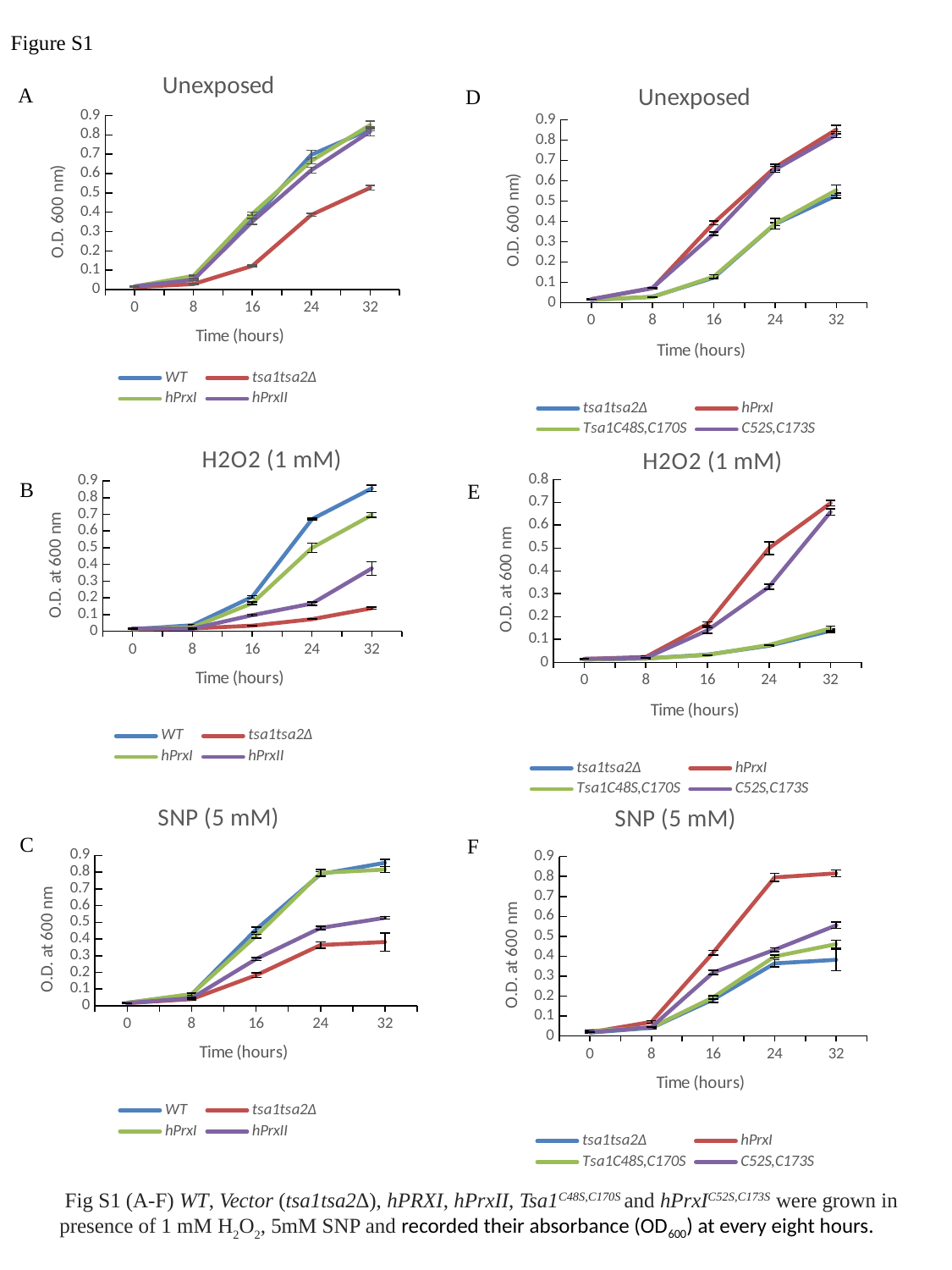

Figure S1
### Chart: Unexposed
| Category | tsa1tsa2Δ | hPrxI | Tsa1C48S,C170S | C52S,C173S |
|---|---|---|---|---|
| 0 | 0.0124 | 0.016390000000000002 | 0.014126666666666668 | 0.016046666666666667 |
| 8 | 0.02854 | 0.07033 | 0.027053333333333332 | 0.07186666666666668 |
| 16 | 0.12238666666666669 | 0.3920666666666666 | 0.12746666666666664 | 0.33863333333333334 |
| 24 | 0.3864666666666667 | 0.6657000000000001 | 0.38786666666666664 | 0.6549666666666667 |
| 32 | 0.5266000000000001 | 0.8513666666666667 | 0.5531666666666667 | 0.8259666666666666 |
### Chart: Unexposed
| Category | WT | tsa1tsa2Δ | hPrxI | hPrxII |
|---|---|---|---|---|
| 0 | 0.015120000000000001 | 0.0124 | 0.016390000000000002 | 0.016396666666666667 |
| 8 | 0.05459666666666666 | 0.02854 | 0.07033 | 0.05078666666666667 |
| 16 | 0.36166666666666664 | 0.12238666666666669 | 0.3920666666666666 | 0.35233333333333333 |
| 24 | 0.6971333333333334 | 0.3864666666666667 | 0.6657000000000001 | 0.6177 |
| 32 | 0.8290666666666667 | 0.5266000000000001 | 0.8513666666666667 | 0.8176 |A
D
### Chart: H2O2 (1 mM)
| Category | tsa1tsa2Δ | hPrxI | Tsa1C48S,C170S | C52S,C173S |
|---|---|---|---|---|
| 0 | 0.011679 | 0.014486666666666667 | 0.01316 | 0.015443333333333335 |
| 8 | 0.016923333333333335 | 0.02347 | 0.016643333333333333 | 0.019039999999999998 |
| 16 | 0.03347666666666666 | 0.16793333333333335 | 0.03215666666666667 | 0.13957 |
| 24 | 0.07238666666666667 | 0.4995 | 0.07606 | 0.33003333333333335 |
| 32 | 0.1380333333333333 | 0.6962 | 0.1484 | 0.6576666666666667 |
### Chart: H2O2 (1 mM)
| Category | WT | tsa1tsa2Δ | hPrxI | hPrxII |
|---|---|---|---|---|
| 0 | 0.012423333333333333 | 0.011679 | 0.014486666666666667 | 0.01705 |
| 8 | 0.03617 | 0.016923333333333335 | 0.02347 | 0.015586666666666665 |
| 16 | 0.20593333333333333 | 0.03347666666666666 | 0.16793333333333335 | 0.09608333333333334 |
| 24 | 0.6713666666666667 | 0.07238666666666667 | 0.4995 | 0.16603333333333334 |
| 32 | 0.8555666666666667 | 0.1380333333333333 | 0.6962 | 0.37646666666666667 |B
E
### Chart: SNP (5 mM)
| Category | WT | tsa1tsa2Δ | hPrxI | hPrxII |
|---|---|---|---|---|
| 0 | 0.017379999999999996 | 0.016086666666666666 | 0.017393333333333334 | 0.0163 |
| 8 | 0.06813999999999999 | 0.03995 | 0.06983 | 0.04576333333333333 |
| 16 | 0.4565666666666666 | 0.18233333333333332 | 0.41741 | 0.28023333333333333 |
| 24 | 0.7883333333333332 | 0.3637066666666667 | 0.7954 | 0.4660666666666667 |
| 32 | 0.8558333333333333 | 0.3819666666666666 | 0.8158666666666666 | 0.5266333333333334 |
### Chart: SNP (5 mM)
| Category | tsa1tsa2Δ | hPrxI | Tsa1C48S,C170S | C52S,C173S |
|---|---|---|---|---|
| 0 | 0.016086666666666666 | 0.017393333333333334 | 0.02427 | 0.017913333333333333 |
| 8 | 0.03995 | 0.06983 | 0.04162 | 0.04392666666666667 |
| 16 | 0.18233333333333332 | 0.41741 | 0.19293333333333332 | 0.3179 |
| 24 | 0.3637066666666667 | 0.7954 | 0.39880000000000004 | 0.43226666666666663 |
| 32 | 0.3819666666666666 | 0.8158666666666666 | 0.4603 | 0.5547 |C
F
 Fig S1 (A-F) WT, Vector (tsa1tsa2∆), hPRXI, hPrxII, Tsa1C48S,C170S and hPrxIC52S,C173S were grown in presence of 1 mM H2O2, 5mM SNP and recorded their absorbance (OD600) at every eight hours.

## Slide 2
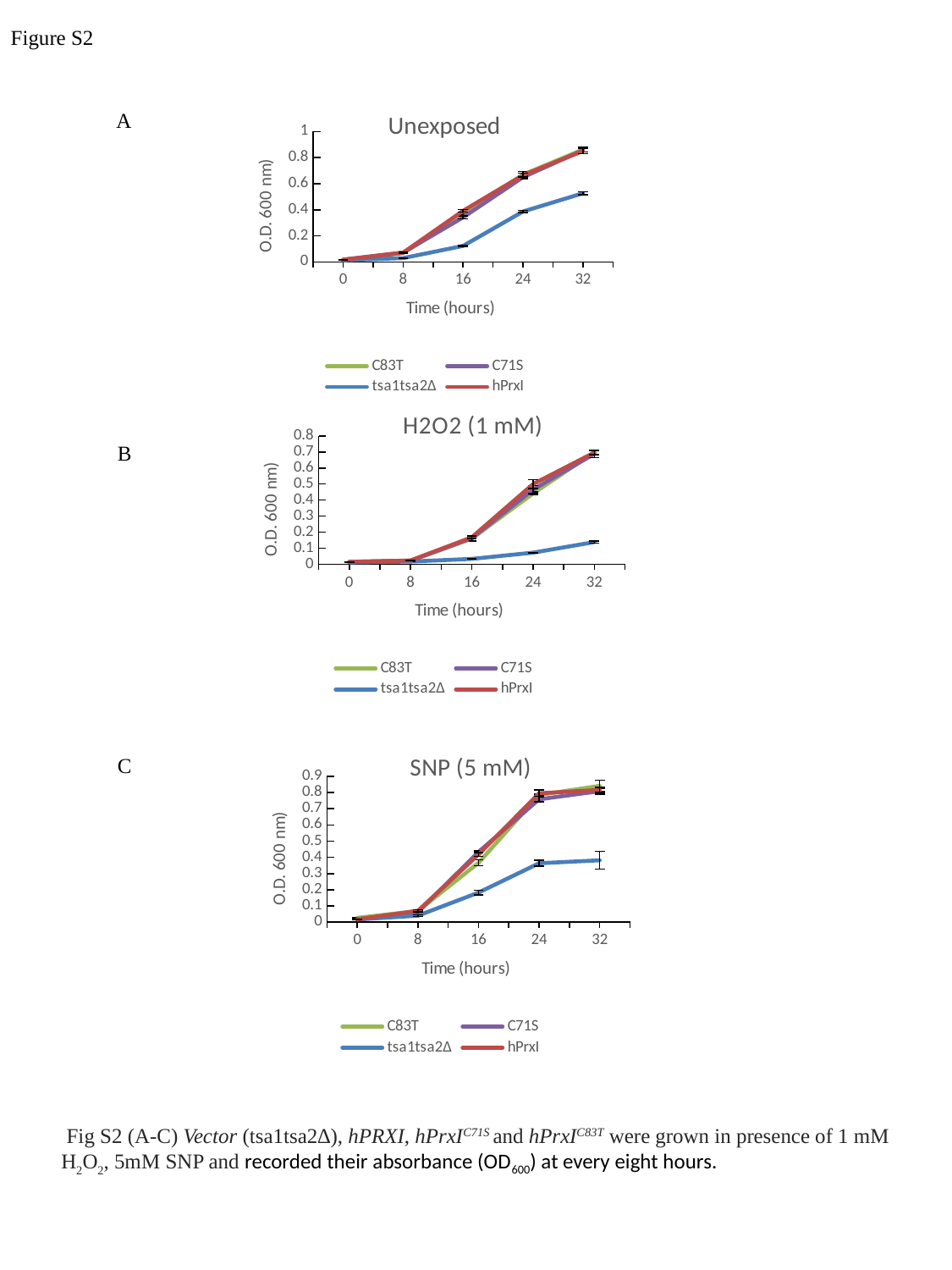

Figure S2
### Chart: Unexposed
| Category | C83T | C71S | tsa1tsa2Δ | hPrxI |
|---|---|---|---|---|
| 0 | 0.014356666666666665 | 0.016583333333333335 | 0.0124 | 0.016390000000000002 |
| 8 | 0.06667666666666666 | 0.07117333333333334 | 0.02854 | 0.07033 |
| 16 | 0.36513333333333337 | 0.3418666666666667 | 0.12238666666666669 | 0.3920666666666666 |
| 24 | 0.6702666666666666 | 0.6501666666666667 | 0.3864666666666667 | 0.6657000000000001 |
| 32 | 0.8624 | 0.8572666666666667 | 0.5266000000000001 | 0.8513666666666667 |A
### Chart: H2O2 (1 mM)
| Category | C83T | C71S | tsa1tsa2Δ | hPrxI |
|---|---|---|---|---|
| 0 | 0.014716666666666668 | 0.015330000000000002 | 0.011679 | 0.014486666666666667 |
| 8 | 0.021986666666666668 | 0.02259 | 0.016923333333333335 | 0.02347 |
| 16 | 0.16028 | 0.16173333333333334 | 0.03347666666666666 | 0.16793333333333335 |
| 24 | 0.44156666666666666 | 0.4656 | 0.07238666666666667 | 0.4995 |
| 32 | 0.6976333333333334 | 0.6880666666666667 | 0.1380333333333333 | 0.6962 |B
### Chart: SNP (5 mM)
| Category | C83T | C71S | tsa1tsa2Δ | hPrxI |
|---|---|---|---|---|
| 0 | 0.026296666666666666 | 0.017 | 0.016086666666666666 | 0.017393333333333334 |
| 8 | 0.06909666666666665 | 0.06131333333333334 | 0.03995 | 0.06983 |
| 16 | 0.3660666666666667 | 0.4333 | 0.18233333333333332 | 0.41741 |
| 24 | 0.7849666666666666 | 0.7582666666666666 | 0.3637066666666667 | 0.7954 |
| 32 | 0.8412666666666667 | 0.8085 | 0.3819666666666666 | 0.8158666666666666 |C
 Fig S2 (A-C) Vector (tsa1tsa2∆), hPRXI, hPrxIC71S and hPrxIC83T were grown in presence of 1 mM H2O2, 5mM SNP and recorded their absorbance (OD600) at every eight hours.

## Slide 3
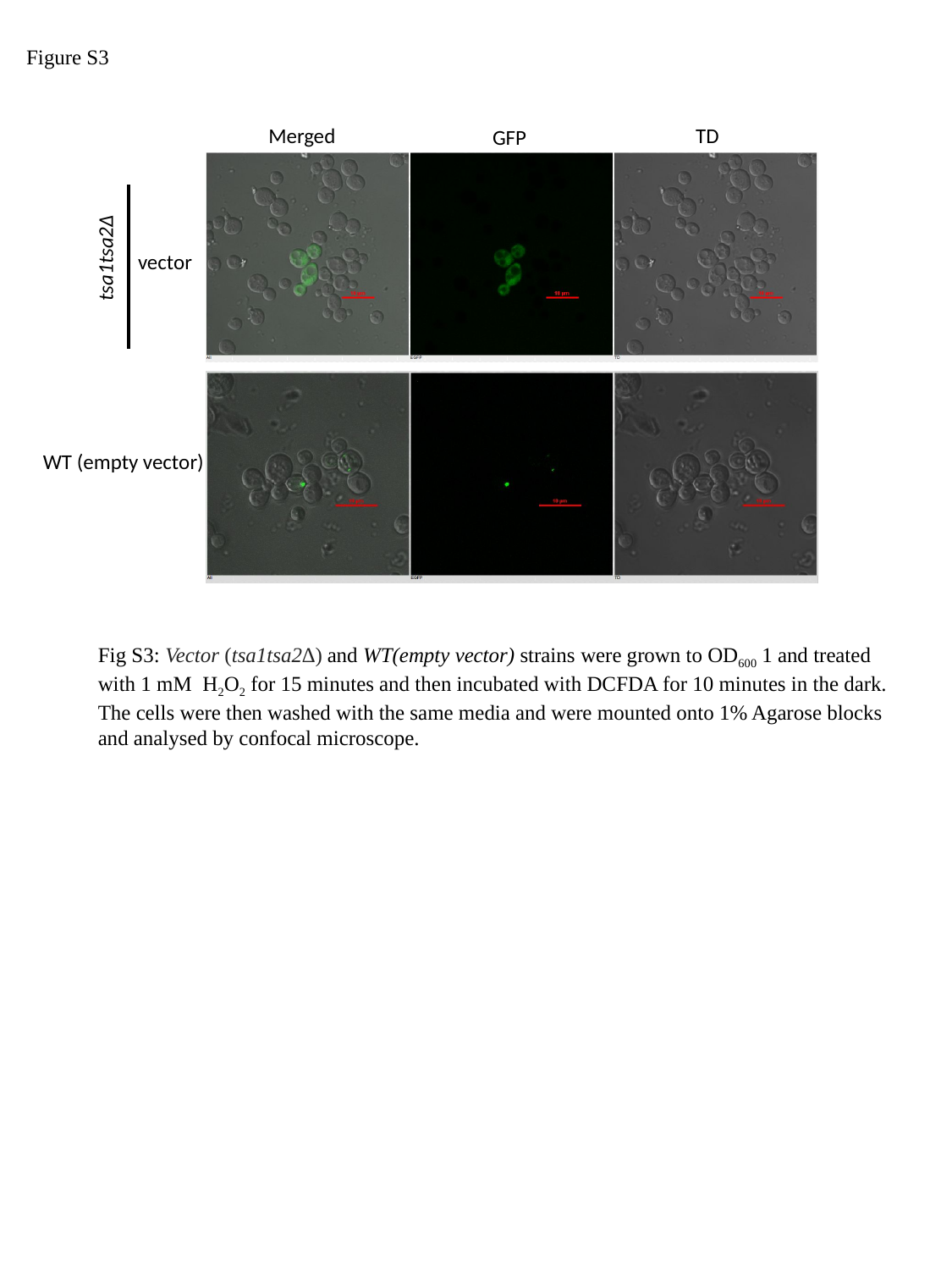

Figure S3
Merged
TD
GFP
tsa1tsa2∆
vector
WT (empty vector)
Fig S3: Vector (tsa1tsa2∆) and WT(empty vector) strains were grown to OD600 1 and treated with 1 mM H2O2 for 15 minutes and then incubated with DCFDA for 10 minutes in the dark. The cells were then washed with the same media and were mounted onto 1% Agarose blocks and analysed by confocal microscope.
